# *Caragana rosea* Turcz Methanol Extract Inhibits Lipopolysaccharide-Induced Inflammatory Responses by Suppressing the TLR4/NF-κB/IRF3 Signaling Pathways

**DOI:** 10.3390/molecules26216660

**Published:** 2021-11-03

**Authors:** Ankita Mitra, Akash Ahuja, Laily Rahmawati, Han Gyung Kim, Byoung Young Woo, Yong Deog Hong, Mohammad Amjad Hossain, Zhiyun Zhang, Soo-Yong Kim, Jongsung Lee, Jong-Hoon Kim, Jae Youl Cho

**Affiliations:** 1 Department of Integrative Biotechnology, Sungkyunkwan University, Suwon 16419, Korea; ankitamitra@skku.edu (A.M.); akashahuja1988@gmail.com (A.A.); lyrahma0106@g.skku.edu (L.R.); hanks523@skku.edu (H.G.K.); 2 AmorePacific R&D Center, Yongin 17074, Korea; quddud@amorepacific.com (B.Y.W.); hydhong@amorepacific.com (Y.D.H.); 3 Department of Veterinary Physiology, College of Medicine, Chonbuk National University, Iksan 54596, Korea; mamjadh2@gmail.com; 4 State Key Laboratory of Systematic and Evolutionary Botany, Institute of Botany, the Chinese Academy of Sciences, Beijing 100093, China; zhangzy@ibcas.ac.cn; 5 International Biological Material Research Center, Korea Research Institute of Bioscience and Biotechnology, Daejeon 34141, Korea; soodole@kribb.re.kr

**Keywords:** *Caragana rosea* Turcz (Cr-ME), anti-inflammatory activity, NF-κB and IRF3 signaling pathways

## Abstract

*Caragana rosea* Turcz, which belongs to the Leguminosae family, is a small shrub found in Northern and Eastern China that is known to possess anti-inflammatory properties and is used to treat fever, asthma, and cough. However, the underlying molecular mechanisms of its anti-inflammatory effects are unknown. Therefore, we used lipopolysaccharide (LPS) in RAW264.7 macrophages to investigate the molecular mechanisms that underlie the anti-inflammatory activities of a methanol extract of *Caragana rosea* (Cr-ME). We showed that Cr-ME reduced the production of nitric oxide (NO) and mRNA levels of iNOS, TNF-α, and IL-6 in a concentration-dependent manner. We also found that Cr-ME blocked MyD88- and TBK1-induced NF-κB and IRF3 promoter activity, suggesting that it affects multiple targets. Moreover, Cr-ME reduced the phosphorylation levels of IκBα, IKKα/β and IRF3 in a time-dependent manner and regulated the upstream NF-κB proteins Syk and Src, and the IRF3 protein TBK1. Upon overexpression of Src and TBK1, Cr-ME stimulation attenuated the phosphorylation of the NF-κB subunits p50 and p65 and IRF3 signaling. Together, our results suggest that the anti-inflammatory activity of Cr-ME occurs by inhibiting the NF-κB and IRF3 signaling pathways.

## 1. Introduction

The immune system comprises many types of cells, such as macrophages, fibroblasts, dendritic cells, and mast cells, to identify invading pathogens using intracellular or surface-expressed pattern recognition receptors (PRRs) [1]. These PRRs, such as toll-like receptors (TLRs), are important for proper functioning of the innate immune response. Among the PRRs, TLR4 is the most studied [2,3]. TLR4 senses bacterial lipopolysaccharide (LPS), and its activation results in nuclear translocation of nuclear factor-κB (NF-κB) and secretion of pro-inflammatory cytokines and chemokines [1,4]. Activation of TLR4 also orchestrates various transcription factors such as interferon regulatory factors 3/7 (IRF 3/7), NF-κB, and activator protein (AP)-1 [5]. IRF3 is part of the response to infection in various cell types [6]. Studies have shown the NF-κB and IRF3 signaling pathways involve the IκB kinase [7,8]. The NF-κB and IRF3 signaling pathways interact with genes at the promotor level [5]. The functionality of the innate immune response relies on cooperation between the NF-κB and IRF3 signaling pathways, which are highly interconnected [9].

The cellular inflammatory response to infection or injury plays a crucial role in host survival and homeostasis [10]. Immune cells such as macrophages, make important contributions to the pathophysiological processes of inflammatory disease [11]. When macrophages are activated by LPS, they use the TLR4 receptor to activate downstream inflammatory signaling through NF-κB, AP-1, and IRF3 [12]. That activation results in translocation of proteins into the nucleus and induces the production of inflammatory cytokines, such as iNOS, interleukin-6 (IL-6), and tumor necrosis factor (TNF)-α, which causes inflammation [13]. Therefore, research focusing on TLR4 signaling has been effective in development of anti-inflammatory drugs. 

In recent years, substantial attention has been given to herbal extracts because they remain a potent source of drug treatments for various diseases. It has been estimated that more than 70% of anti-cancer and anti-inflammatory drugs originate from natural herbal extracts [14,15,16]. *Caragana rosea* Turcz, belonging to the Leguminosae family, is a small shrub found in Northern and Eastern China. It contains maackin, scirpusin A, scirpusin B, *cis*-scirpusin, and stilbene dimers [16]. Scirpusin B is active against HIV [16,17]. Moreover, the root of the plant has significant anti-inflammatory activity against conditions such as fever, asthma, and cough [16,18]. However, the underlying mechanisms of its anti-inflammatory effects have not been elucidated. Based on the traditional remedies that use *Caragana rosea*, we hypothesized that *Caragana rosea* methanol extract (Cr-ME) would protect cells from injuries induced by LPS. To test that idea, we investigated the mechanisms of Cr-ME and evaluated its anti-inflammatory properties using stimulated inflammatory responses. Our results confirm that Cr-ME inhibits inflammation in LPS-stimulated macrophages by suppressing NF-κB and IRF3 signaling.

## 2. Results

### 2.1. Effects of Cr-ME on Nitric Oxide (NO) Level and Cell Viability in LPS-Treated RAW264.7 Macrophages

**Figure 1:**
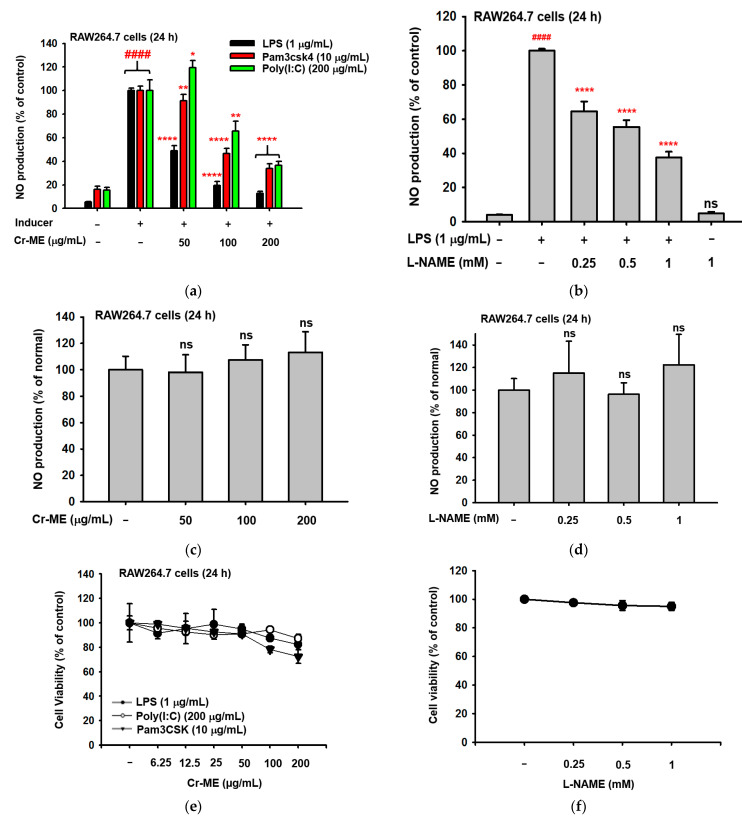

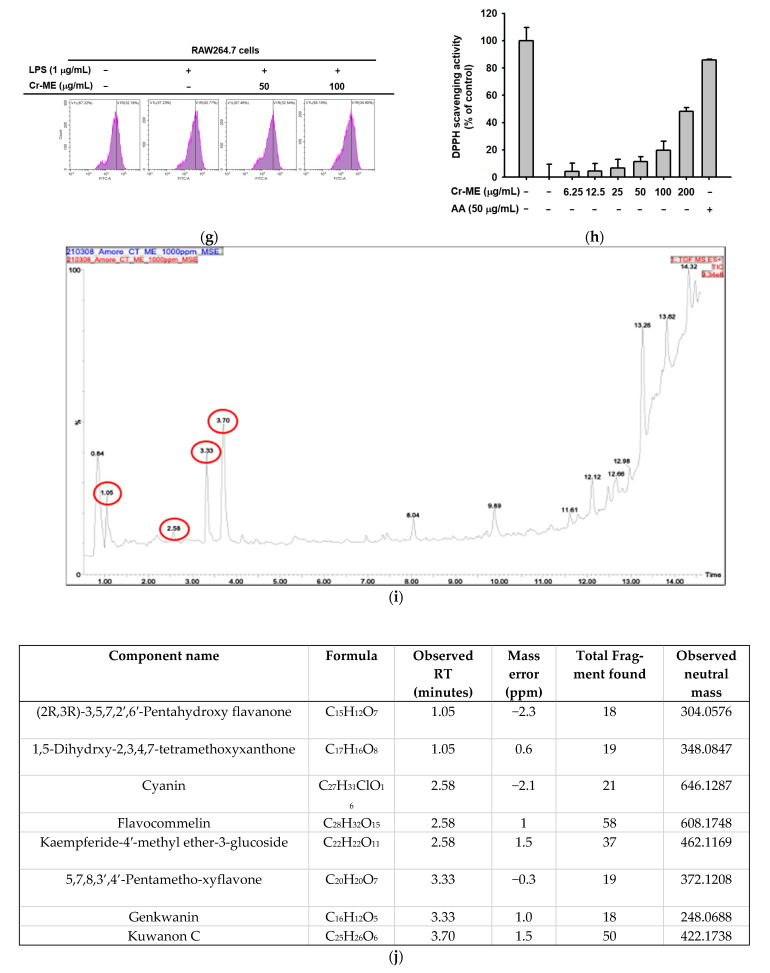
Effects of Cr-ME on NO production in macrophages. (**a**–**d**) The levels of NO production in RAW264.7 cells stimulated with (**a**,**b**) or without (**c**,**d**) LPS (1 μg/mL), pam3CSK (10 μg/mL) or poly(I:C) (200 μg/mL) in the presence or absence of Cr-ME (**a**,**c**) or L-NAME(**b**,**d**) for 24 h were measured by Griess assay. (**e**,**f**) The viabilities of RAW264.7 cells treated with the indicated doses of Cr-ME (**e**) or L-NAME (**f**) for 24 h were determined using the MTT assay. (**g**) Treatment with Cr-ME (50 and 100 μg/mL) decreased ROS generation by 32.54% and 34.85%, respectively, compared with LPS-stimulated cells. (**h**) DPPH radical scavenging activity was employed to check antioxidative activity of Cr-ME (100 to 200 μg/mL). (**i**,**j**) The major polyphenols of Cr-ME were identified by LC-MS/MS analysis. Representative histogram (**i**) and components (**j**) are summarized. All the data (**a**–**f**,**h**) are expressed as the mean ± SD of three independent experiments. Statistical significance was calculated using one-way ANOVA (Dunnett’s *t*-test). ns: Not significant. #### *p* < 0.0001 compared to the normal group and * *p* < 0.05, ** *p* < 0.01, and **** *p* < 0.0001 compared to the pam3CSK, poly(I:C), and LPS groups.

To evaluate whether Cr-ME suppresses secreted inflammatory mediators during an inflammatory response, we examined NO production in RAW264.7 macrophages treated with different concentrations of Cr-ME in the presence or absence of LPS, poly(I:C), and pam3csk for 24 h. Interestingly, Cr-ME dose-dependently suppressed LPS-, poly(I:C)-, and pam3csk-mediated NO production in RAW264.7 macrophages, as shown in the case of L-NAME (Figure 1a,b), a standard compound known to block iNOS [19]. In contrast, there was no induction or inhibition of NO by Cr-ME or L-NAME under no LPS-treated conditions (Figure 1c,d). In addition, Cr-ME (50, 100, and 200 μg/mL) and L-NAME (0.25, 0.5, and 1 mM) did not reduce the viability of RAW264.7 cells at (Figure 1e,f). Interestingly, the 100 μg/mL concentration of Cr-ME showed the lowest NO production and highest cell viability in the presence of LPS, poly(I:C), and pam3csk. Therefore, in subsequent experiments, we treated RAW264.7 cells with 100 μg/mL concentration. 

Next, we evaluated the effect of Cr-ME on ROS production in LPS-treated RAW264.7 macrophages. After 24 h of LPS treatment, we evaluated the intracellular ROS levels in the macrophages. We observed that Cr-ME treatment significantly and dose-dependently reduced the LPS-induced ROS level in macrophages. Treatment with 50 μg/mL and 100 μg/mL Cr-ME decreased ROS generation by 32.54% and 34.85%, respectively, compared with the LPS-stimulated control cells (Figure 1g). Therefore, Cr-ME also has antioxidant effects in RAW264.7 cells. In addition, we evaluated the scavenging activity of Cr-ME using the DPPH radical scavenging assay with ascorbic acid as the reference. Those results showed that Cr-ME showed good scavenging activity even at low concentration (Figure 1h).

In addition, LC-MS/MS analysis has been performed in order to characterize the phenolic composition in the Cr-ME (Figure 1i). The major polyphenolic components in Cr-ME were (2R,3R)-3,5,7,2′,6′-pentahydroxyflavanone (1.05), 1,5-dihydroxy-2,3,4,7-tetramethoxyxanthone (1.05), cyanin (2.58), flavocommelin (2.58), kaempferide-4′-methyl ether-3-glucoside (2.58), 5,7,8,3′,4′-pentametho-xyflavone (3.33), genkwanin (3.33), and kuwanon C (3.77), as summarized in Figure 1j.

### 2.2. Effects of Cr-ME on Inflammatory Gene Expression at the Transcriptional Level

**Figure 2:**
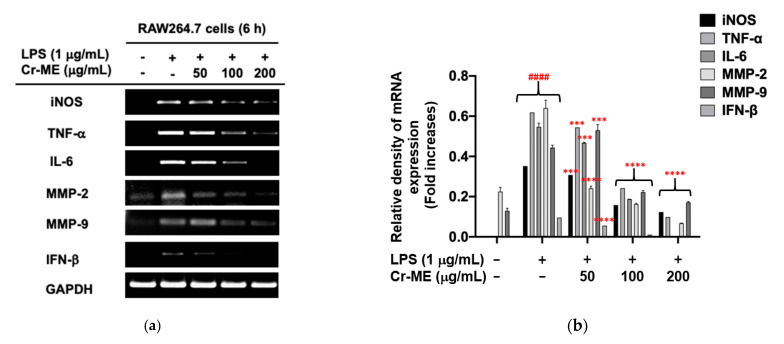

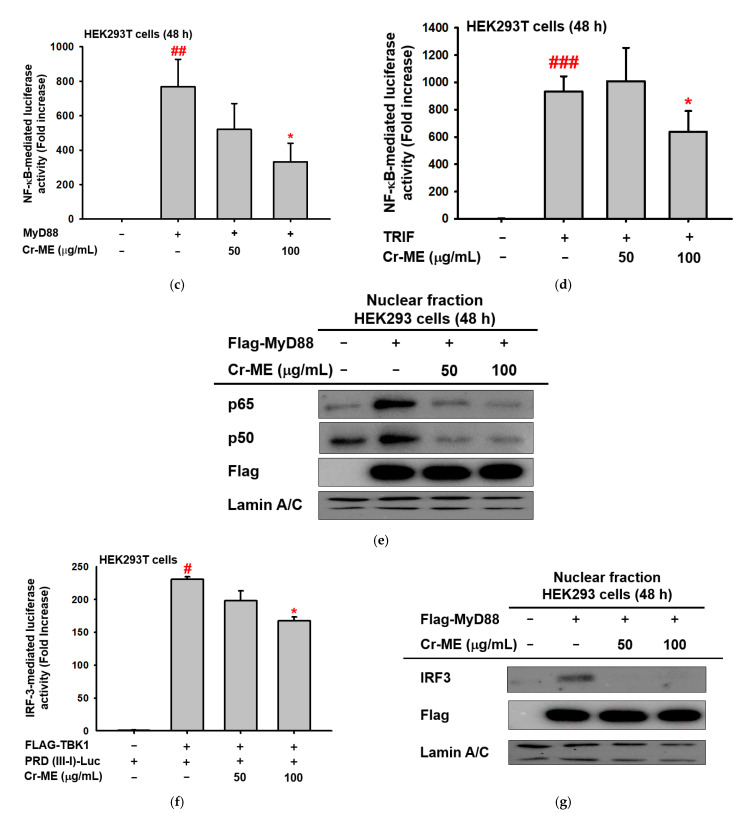

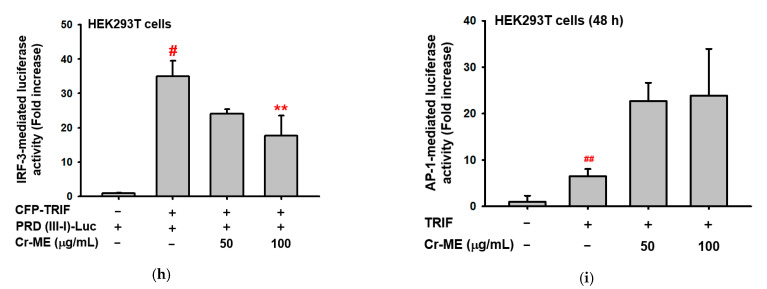
Suppressive effect of Cr-ME on the mRNA expression of inflammatory genes and their transcriptional activation. (**a**,**b**) The mRNA levels of inflammatory genes from RAW264.7 cells-stimulated with LPS (1 μg/mL) for 6 h in the presence or absence of Cr-ME (5 to 200 μg/mL) were measured by semi-quantitative RT-PCR (**a**) and relative band intensity of inflammatory genes was quantified using ImageJ software (**b**). (**c**,**d**,**f**,**h**,**i**) Effect of Cr-ME on transcription factor activation was determined by luciferase assay. HEK293T cells overexpressed with FLAG-MyD88 (**c**), CFP-TRIF (**d**,**h**,**i**) or FLAG-TBK1 (**f**) were transfected with plasmid constructs of NF-κB-Luc (**c**,**d**), PRD (III-I)-Luc (**f**,**h**), AP-1-Luc (**i**), and β-gal (as a transfection control) for 24 h, followed by treatment with Cr-ME (50–100 μg/mL) for additional 24 h. Luciferase activity was measured using a luminometer and normalized to that of β-gal. (**e**,**g**) Levels of p65, p50, Flag, IRF3, and lamin A/C in the nuclear fraction of HEK293T cells co-transfected with MyD88 (**e**) or TBK1 (**g**) were determined by Western blotting analysis. All the data (**b**–**d**,**f**,**h**,**i**) are expressed as the mean ± SD of three independent experiments. Relative band intensity (**b**) was measured using ImageJ. Statistical significance was calculated using one-way ANOVA (Dunnett’s *t*-test**).** # *p* < 0.05, ## *p* < 0.01, ### *p* < 0.001, and #### *p* < 0.0001 compared with the normal group, *** *p* <0.001 and **** *p* < 0.0001 compared to the LPS group, * *p*< 0.05 and ** *p* < 0.01 compared to control group.

To explore whether Cr-ME-guided suppression of NO production is regulated at the transcriptional level, we examined the mRNA expression of genes responsible for inflammation. RAW264.7 macrophages were treated with Cr-ME for 30 min (50, 100, and 200 μg/mL) and then stimulated with LPS for six hours. The mRNA expression was examined using semi-quantitative RT-PCR. This analysis showed that the gene expression levels of iNOS, TNF-α, IL-6, MMP-2, MMP-9, and IFN-β decreased dose-dependently in the presence of Cr-ME (Figure 2a,b). Overall, these results correspond with our finding about NO production level and indicate that Cr-ME suppresses LPS-induced NO production level by downregulating iNOS gene expression. The NF-κB and IRF3 signaling pathways are known to drive the production of pro-inflammatory cytokines and regulate cell proliferation, apoptosis, and inflammation [20,21]. Because we observed that Cr-ME suppressed the mRNA levels of pro-inflammatory cytokines, we performed an NF-κB/IRF3-mediated luciferase reporter gene assay using HEK293T cells to investigate whether Cr-ME inhibited the translocation involved in the NF-κB and IRF3 signaling pathways. We co-transfected cells with MyD88, an adaptor molecule of NF-κB signaling, and TBK1, a signaling molecule in IRF3 activation, and then treated them with Cr-ME (50 and 100 μg). Those results show a significant dose-dependent reduction in MyD88-, TRIF-, and TBK1-mediatd luciferase activity (Figure 2c,d,f,h). Thus, Cr-ME modulates its anti-inflammatory activity through MyD88 and TBK. To confirm whether suppression of NF-κB and IRF3 activities by Cr-ME is due to blockade of nuclear translocation of these proteins, we also determined the nuclear levels of these transcription factors. As Figure 2e,g show, expectedly, the nuclear levels of NF-κB and IRF3 were reduced by Cr-ME. We also evaluated an AP-1-mediated luciferase reporter assay to determine whether Cr-ME inhibited the translocation of AP-1 in the NF-κB and IRF3 signaling pathways. However, the results of that test did not show a dose-dependent reduction in MyD88 and TBK1 adaptor proteins (Figure 2i). Therefore, Cr-ME targets the MyD88 and TBK1 adaptor proteins in the NF-κB and IRF3 signaling pathways.

### 2.3. Effects of Cr-ME on LPS-Induced NF-κB Signaling and Its Upstream Enzyme Src Activity

**Figure 3:**
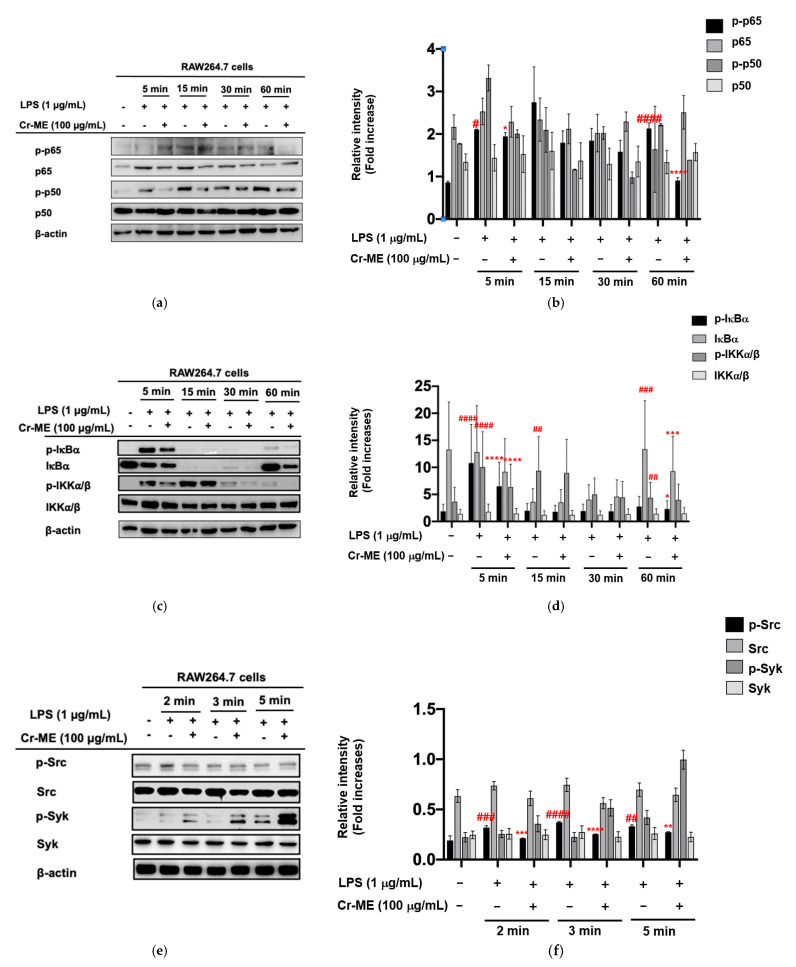

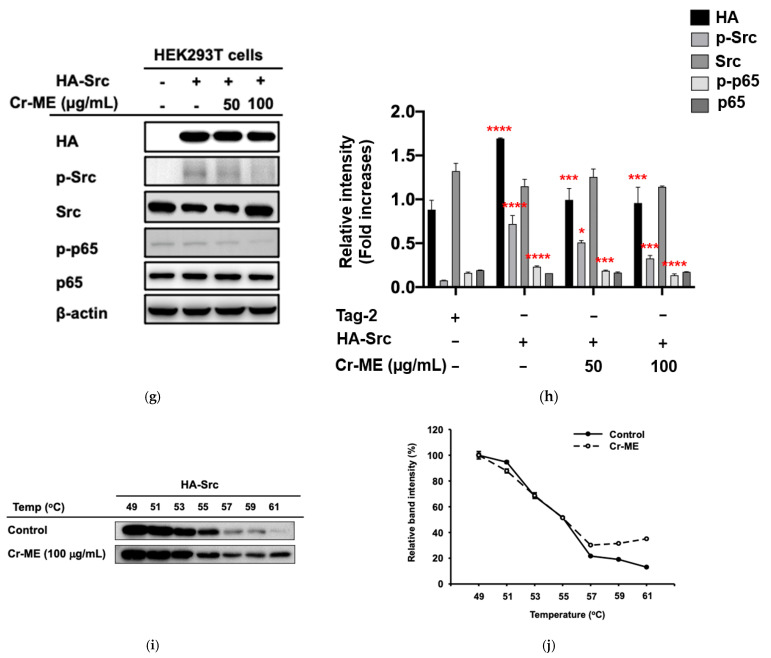
Effect of Cr-ME on NF-κB pathway and its upstream enzyme Src activation. (**a**,**b**) The levels of phospho- forms of NF-κB subunits (p-p65 and p-p50) and their total proteins were determined by Western blotting analysis with whole cell lysates of RAW264.7 cells treated with LPS (1 μg/mL) in the presence or absence of Cs-ME (100 μg/mL) for the indicated times (**a**). Relative intensity of these proteins was calculated by ImageJ (**b**). (**c**,**d**). The levels of phospho-forms of IκBα and IKKα/β and their total proteins were determined by Western blotting analysis with whole cell lysates of RAW264.7 cells treated with LPS (1 μg/mL) in the presence or absence of Cs-ME (100 μg/mL) for the indicated times (**c**). Relative intensity of these proteins was calculated by ImageJ (**d**). (**e**,**f**) The levels of phospho-forms of Src and Syk and their total proteins were determined by Western blotting analysis with whole cell lysates of RAW264.7 cells treated with LPS (1 μg/mL) in the presence or absence of Cs-ME (100 μg/mL) for the indicated times (**e**). Relative intensity of these proteins was calculated by ImageJ (**f**). (**g**,**h**) The levels of phospho-forms of Src and p65 and their total proteins were determined by Western blotting analysis with whole cell lysates of HEK293T cells were transfected HA-Src in the presence or absence of Cs-ME (100 μg/mL) for 24 h (**g**). Relative intensity of these proteins was calculated by ImageJ (**h**). (**i**,**j**) The CETSA assay was conducted in the HA-Src-transfected HEK293T cells treated with Cr-ME (100 μg/mL) or DMSO (as a control) and then, Src level was determined by Western blotting analysis (**i**). Relative intensity of Src was calculated by ImageJ (**j**). All the data (**b**,**d**,**f**,**h**,**j**) expressed as the mean ± SD of three independent experiments. Statistical significance was calculated using one-way ANOVA (Dunnett’s *t*-test). # *p* < 0.05, ## *p* < 0.01, ### *p* < 0.001, and #### *p* < 0.0001 compared to normal group, and * *p* < 0.05, ** *p* < 0.01, *** *p* < 0.001, and **** *p* < 0.0001 compared to the control group.

Because our previous results showed significant suppression of NF-κB luciferase reporter gene activity under Cr-ME stimulation, we used Western blotting analysis to investigate the intracellular signaling components of the NF-κB (Figure 3a,b). We found that Cr-ME strongly suppressed the LPS-induced increase in phosphorylation of IKKα/β, a major subunit of NF-κB signaling after 5 min (Figure 3c,d), implying that upstream signaling events could be the potential molecular targets of Cr-ME. We then tested the inhibitory activity of Cr-ME on Syk and Src, NF-κB upstream signaling kinases at 2, 3, and 5 min. Interestingly, Cr-ME treatment clearly restored the phosphorylation level of Src at 2, 3, and 5 min after LPS treatment, whereas the Syk kinase did not change significantly upon Cr-ME treatment (Figure 3e,f). We then performed Src overexpression experiments in HEK293T cells to confirm the inhibitory effects of Cr-ME. Cr-ME (50–100 μg) treatment significantly and dose-dependently reduced the phosphorylation levels of Src and p65 (Figure 3g,h). Our results suggest that Cr-ME targets the Src protein kinase with respect to NF-κB signaling.

In addition, we also performed the CETSA to determine whether Cr-ME interacts directly with Src in vitro and to verify the interaction between the target protein and test compound through ligand-mediated stabilization of proteins [22,23]. In the CETSA, cells with and without the test compound are damaged thermally to see whether binding between the compound and the target produces thermostabilization of the target [24]. The thermal stability of Src in Src-overexpressed HEK293T cells was confirmed from 35 to 71 °C after 24 h of Cr-ME pre-treatment. In the results, the Src protein was distinctly detectable in the Cr-ME-treated group but not in the control group (Figure 3i,j), confirming direct interaction between Cr-ME and Src in HEK293T cells.

### 2.4. Effects of Cr-ME on LPS-Induced IRF3 Signaling and Its Upstream Enzyme TBK1 Activity

**Figure 4:**
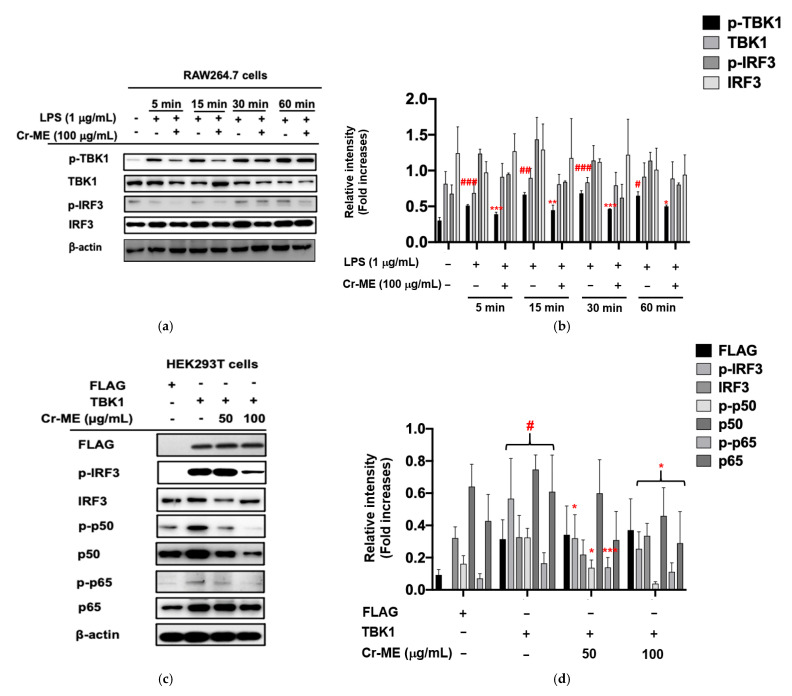
Effect of Cr-ME on the IRF3 pathway and its upstream enzyme TBK1 activation. (**a**,**b**) RAW264.7 cells pretreated with 100 μg/mL of Cr-ME were stimulated by LPS (1 μg/mL) for the indicated times. Then, the phosphorylation levels of TBK1 and IRF3 were detected by Western blotting analysis (**a**). Relative intensity of these proteins was calculated by ImageJ (**b**). (**c**,**d**) HEK293T cells were transfected with TBK1 along with Cr-ME (50 and 100 μg/mL). Then, the phosphorylation levels of IRF3, p50, and p65 were determined by Western blotting analysis (**c**). Relative intensity of these proteins was calculated by ImageJ (**d**). All the data (**b**,**d**) expressed as the mean ± SD of three independent experiments. Statistical significance was calculated using one-way ANOVA (Dunnett’s *t*-test). # *p* < 0.05, ## *p* < 0.001, and ### *p* < 0.001 compared to normal group, and * *p* < 0.05, ** *p* < 0.005, and *** *p* < 0.001 compared to control group.

Based on our previous findings [8,25], we hypothesized that TBK1 protein targets for the anti-inflammatory activity of Cr-ME. In addition, our previous results showed significant suppression of IRF-3 luciferase reporter gene activity under Cr-ME stimulation Therefore, we performed Western blotting analysis to determine the intracellular signaling components of the IRF3 pathways. We observed that LPS increased IRF3 phosphorylation and Cr-ME suppressed this phosphorylation (Figure 4a,b), implying that upstream signaling events could be the potential molecular targets of Cr-ME. Interestingly, we found that TBK1 phosphorylation was time-dependently increased from 5 to 60 min and its phosphorylation at 5 and 15 min was also reduced by Cr-ME (Figure 4a,b), suggesting that TBK1 could be the molecular target of Cr-ME in relation to IRF3 signaling. We then performed TBK1 overexpression experiments in HEK293T cells to confirm the inhibitory effects of Cr-ME. Cr-ME (50–100 μg) treatment significantly and dose-dependently reduced the phosphorylation levels of p65 (Figure 4c,d). We observed that TBK1 overexpression significantly reduced the phosphorylation of IRF3, p50, and p65 in the Cr-ME groups (Figure 4c,d). These results indicate that Cr-ME directly targets TBK1 activity in the production of its anti-inflammatory activity. Taken together, our data suggest that Cr-ME exerts anti-inflammatory effects by controlling IRF3 signaling pathways.

### 2.5. Effects of Cr-ME Treatment on LPS-Induced Acute Lung Injury (ALI)

Because we observed Cr-ME attenuation of inflammation in vitro at concentrations of 50 and 100 μg/mL, we further evaluated its anti-inflammatory effects in an LPS-induced model of acute lung injury in mice by considering the Cr-ME at the same concentration (50 and 100 mg/kg).

**Figure 5:**
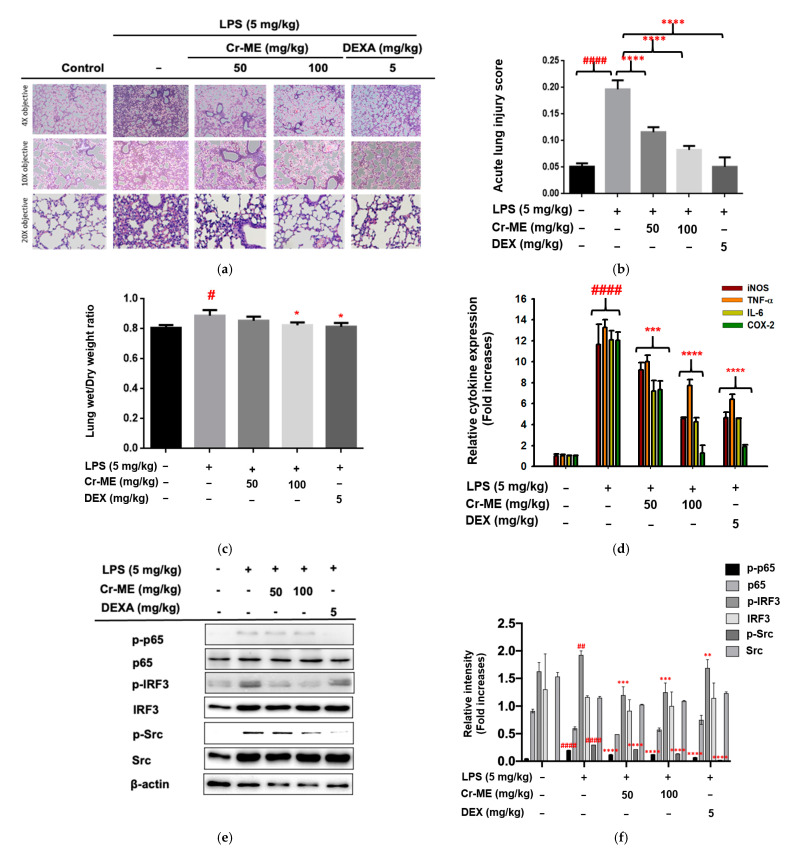
Effect of Cr-ME treatment on LPS-induced acute lung injury (ALI). (**a**,**b**) Histological analysis was performed to visualize the inhibitory activity of Cr-ME in LPS-induced acute lung injury conditions of mice after 16 h of LPS instillation (**a**). H&E stain was applied to the sections, original magnification, 200×. Acute lung injury scores were calculated according to parameters indicated in Table 1 (**b**). (c) The effect of Cr-ME on pulmonary edema was determined by calculating the lung wet/dry weight ratio. (**d**) The mRNA expression levels of inflammatory genes were determined by real-time PCR. (**e**,**f**) The total and phospho-forms of p65, IRF3, Src, and β-actin were analyzed by Western blotting analysis performed with tissue lysates from the LPS-induced ALI mice (**e**). Relative intensity of these proteins was calculated by ImageJ (**f**). All the data (**b**–**d**,**f**) expressed as the mean ± SD of 7 mice or three independent experiments. Relative band intensity was measured using ImageJ Values are mean ± SD (*n* = 5 per group). Statistical significance was calculated using one-way ANOVA (Dunnett’s *t*-test). # *p* < 0.05, ## *p* < 0.001, and #### *p* < 0.0001 compared to normal group, * *p* < 0.05, ** *p* < 0.01, *** *p* < 0.001, and **** *p* < 0.0001 compared to LPS group.

First, we examined histological changes in the lung tissues of normal and LPS-induced ALI mice after 16 h of treatment with saline, Cr-ME, and LPS. The control group showed normal pulmonary histology, whereas the lungs from the mice treated with LPS showed pathological changes: cell infiltration, interstitial edema, cell clumping, alveolar hemorrhage, and thickening of the alveolar wall (Figure 5a). Those morphological changes were significantly smaller in the LPS-induced ALI mice treated with Cr-ME (50 and 100 mg/kg) or DEXA (5 mg/kg). These results imply that Cr-ME has very strong potential to attenuate LPS-induced lung inflammation, decrease the level of morphological changes, and weaken the effects of alveolar hemorrhaging during ALI. Similarly, the lung injury scores of the Cr-ME groups calculated according to the parameters indicated in Table 1 were significantly lower than those of the LPS group (*p* < 0.0001) (Figure 5b). We also measured the effect of Cr-ME treatment on the lung wet/dry ratio 16 h after LPS instillation. As shown in Figure 5c, we found a significant difference in the lung wet/dry ratio among the LPS, Cr-ME, DEXA, and control groups (*p* < 0.01, respectively). A substantial increase in the lung wet/dry weight ratio was observed in the LPS group compared with the PBS group (*p* < 0.01). However, compared with the LPS group, the lung wet/dry weight ratio decreased significantly in the animals treated with Cr-ME (100 mg/kg) and DEXA (5 mg/kg) after LPS challenge (*p* < 0.01 for both).

Furthermore, the mRNA levels of the pro-inflammatory genes TNF-α, IL-6, iNOS, and COX-2 in the lung tissues of LPS-induced ALI mice were found to increase significantly compared with the control group (*p* < 0.0001) (Figure 5d). However, a significant downregulation of those mRNA levels was observed in the Cr-ME groups (50 and 100 mg/kg) and the DEXA (5 mg/kg) group compared with the LPS group (*p* < 0.0001 for all). Finally, to determine whether the phosphorylation of NF-κB, p65, IRF3, and Src in murine lung tissues is reduced, Western blotting analysis was performed. As demonstrated in Figure 5e,f, mice challenged with LPS showed significantly increased expression and activation of NF-κB, IRF3, and Src in their lung tissue compared with the control group. However, Cr-ME treatment markedly inhibited NF-κB, IRF3, and Src activation, as assessed by measuring their phosphorylation levels, compared with the LPS-treated mice. Therefore, Cr-ME has the potential to inhibit the TLR4-mediated NF-κB, IRF3, and Src signaling pathway.

## 3. Discussion

Inflammatory damage contributes to the progression of chronic inflammatory diseases. It can induce the production of inflammatory mediators such as chemokines and cytokines. Many natural herb derived extracts used in Chinese traditional medicine have been reported as anti-inflammatory drugs that can be used to treat diseases such as colitis and gastritis [22,23]. Cr-ME is derived from a small shrub with anti-inflammatory properties found in Northern and Eastern China, and it has proven to be effective in treating models of fever, asthma, and cough [16,18]. The history of Cr-ME as an important traditional folk medicine motivated us to study its underlying molecular mechanisms in inflammatory conditions. Therefore, we explored the anti-inflammatory effects of Cr-ME in vitro using LPS stimulation in macrophages and in vivo in an LPS-induced ALI model in mice.

Our results show that Cr-ME dose-dependently diminishes the NO production induced in macrophages by LPS, pam3CSK, and poly(I:C) stimulation without influencing cell viability (Figure 1a,e). Previous reports have shown that overproduction of NO and PEG2 in macrophages promotes inflammation and immune disorders [26,27,28]. Therefore, the downregulation of NO production caused by Cr-ME suggests that this natural compound could be used in the treatment of inflammatory disease. We also investigated pro-inflammatory cytokines at the transcriptional level in LPS-stimulated RAW264.7 cells. Those results clearly show that Cr-ME significantly suppresses the expression of iNOS, IL-6, TNF-α, IFN-β, and MMP-2/-9. Previous studies reported that the key enzyme responsible for NO production is iNOS [15], and we found that Cr-ME significantly suppresses iNOS gene expression in LPS-stimulated RAW264.7 macrophages (Figure 2a). In addition, oral administration of Cr-ME in a murine model of ALI decreased pulmonary edema (as shown by lung weight) and injury compared with the LPS only group (Figure 5a–c). Furthermore, we evaluated the mRNA levels of pro-inflammatory cytokines in LPS-induced ALI mice. Consistent with our in vitro data, we observed that Cr-ME significantly decreased pro-inflammatory cytokines (iNOS, IL-6, TNF-α, and COX-2) compared with level in the LPS only group (Figure 5d).

LPS-induced inflammation is known to activate the NF-κB, MAPK, and IRF3 signaling pathways in macrophages [29,30]. Activation of intracellular inflammatory signaling also regulates cytokine production, cell proliferation, and activation and regulation of inflammation and the immune response [31,32]. The TLR4 signaling pathway is a novel therapeutic target for the treatment of inflammatory disease [33]. TLR4 signaling activation initiates the release of pro-inflammatory cytokines and chemokines such as TNF-α, IL-6, and IL-1β [34]. Our results show that Cr-ME effectively and dose-dependently downregulated the overproduction of TNF-α and IL-6 induced by LPS treatment, indicating that Cr-ME has an inhibitory role at the transcriptional level (Figure 2a,b). Reports have shown that TLR4 activation by LPS requires MyD88, TRIF, or TRAM to instruct the Src, TBK1, and IKKε kinases to activate NF-κB and IRF3 signaling [35]. Our findings indicate that Cr-ME dose-dependently inhibited NF-κB and IRF3 luciferase activity (Figure 2), which implies that it can inhibit the upstream signaling of NF-κB and IRF3.

Upon activation, TLR4 enrolls the intracellular proteins MyD88 and TRIF and activates IKK complex proteins (IKKα, IKKβ, and IKKγ) [36]. During that process, NF-κB/ p65 complexes are released from the cytoplasm and translocate to the nucleus. We found that Cr-ME time-dependently reduced the phosphorylation of IKKα/β and IκBα in LPS-treated RAW264.7 macrophages (Figure 3c,d), indicating that Cr-ME suppressed the NF-κB signaling pathway. Indeed, both active forms of p65 and p50 and the nuclear levels of these proteins were also found to be inhibited by Cr-ME (Figure 2e and Figure 3a). Previously, our group reported that the upstream protein kinases Syk and Src play an important role in regulating NF-κB signaling [24,25]. Therefore, we studied upstream Syk and Src signaling events early after LPS stimulation. We observed that phosphorylation of Syk and Src increased time-dependently in the Cr-ME treatment groups (Figure 3e,f). Thus, our overall findings suggest that Cr-ME targets Syk or Src in NF-κB signaling (Figure 3). In addition to NF-κB signaling, the TBK1/IRF3 signaling pathway, which corresponds to LPS-induced inflammation via TBK1, can phosphorylate IRF3 and promote its nuclear translocation [35]. Our data show that levels of phospho-IRF3 and nuclear IRF3 were time-dependently decreased in the Cr-ME treated groups (Figure 2g and Figure 4a,b), whereas the upstream TBK1 increased time-dependently (Figure 4a,c), indicating that Cr-ME targets TBK1 with respect to IRF3 signaling. Similarly, in the lungs of mice with LPS-induced ALI, we also observed that Cr-ME inhibited NF-κB and IRF3 protein expression compared with that in the LPS only group (Figure 5e,f).

The Src tyrosine kinase is associated with a non-receptor membrane protein known to be active in carcinoma tissues [37]. Several publications agree that 70% of inflammatory and oncogenic processes code for that tyrosine kinase [38]. Moreover, overactivation of Src indicates chemoresistance, and Src inhibition can reverse chemoresistance in many cancer cells [39]. We next tested the overexpression of Src and TBK1 in HEK293T cells and examined the signaling molecules downstream of NF-κB in the presence of Cr-ME. Src overexpression highly phosphorylated Src and p65, and Cr-ME dose-dependently downregulated that phosphorylation (Figure 3g,h). Interestingly, similar results were observed with TBK1 overexpression, which caused high phosphorylation of IRF-3, p65, and p50 that was dose-dependently suppressed by Cr-ME (Figure 4c,d). Those results indicate that Cr-ME relieved LPS-induced inflammation by downregulating the activation of the TLR4-NF-κB-IRF3 signaling pathways (Figure 3 and Figure 4). The CETSA results confirm the direct interaction between Cr-ME and Src in HEK293T cells (Figure 3i,h), which implies that Cr-ME specifically targets the Src kinase. Overall, our results suggest that the anti-inflammatory action of Cr-ME helps to block Src in the NF-κB upregulation cascade. Consistent with those in vitro results, we found that Cr-ME significantly downregulates Src protein expression in the lungs of mice with LPS-induced ALI (Figure 5e,f).

Considering the results of LC-MS/MS analysis of Cr-ME, (2*R*,3*R*)-3,5,7,2′,6′-pentahydroxy flavanone (1.05), 1,5-dihydroxy-2,3,4,7-tetramethoxyxanthone (1.05), cyanin (2.58), flavocommelin (2.58), kaempferide-4′-methyl ether-3-glucoside (2.58), 5,7,8,3′,4′-pentamethoxyflavone (3.33), genkwanin (3.33), and kuwanon C (3.77) are the major polyphenols of Cr-ME, known as anti-inflammatory flavonoids [40,41]. (Figure 1i,j). Previously, a study reported that (2*R*,3*R*)-3,5,7,2′,6′-pentahydroxy flavanone has a significant anti-arthritic activity effect in rat by modulating the inflammatory pathway [42]. The other major polyphenols which we observed in Cr-ME is 1,5-dihydrxy-2,3,4,7-tetramethoxyxanthone which is mainly presents in most plants and known to have potent anti-inflammatory effect [43]. In addition, it was found that xanthone can act as vasodilator [44]. Next, cyanin, also known as cyanin chloride or cyanidin-3,5-di-O-glucoside, was reported to induce apoptosis by inhibiting NF-κB signaling through activation of Nrf2 in colorectal cancer cells [45]. Flavocommelin, which is one of the components identified in *Commelina communis* [46] and was reported to reduce lung damage in influenza virus-infected mice [47]. Regarding kaempferide-4′-methyl ether-3-glucoside, it is considered as a valuable functional food ingredient with a broad range of therapeutic applications such as anti-cancer, antioxidant, and anti-inflammatory uses [48]. Other major component, 5,7,8,3′,4′-pentametho-xyflavone, also known as isosinensetin, was revealed to display higher antioxidant and anti-inflammatory activities [49]. For genkwanin, researchers reported that it possesses anti-inflammatory activity mainly through the regulation of the miR-101/MKP-1/MAPK pathway in vitro [50] and has antitumor activity against cancer [51]. Kuwanon C is neuroprotective and anti-inflammatory compound by modulating HO-1 expression and Nrf2 expression [52]. Therefore, we can infer that Cr-ME with these major polyphenols and anti-inflammatory components can possess therapeutic potential against various inflammatory diseases such as arthritis, gastritis, and hepatitis. Indeed, it was reported that this plant has good effects against some inflammatory symptoms such as cough, fever, and asthma [18]. Although we did not directly test the therapeutic efficacy of Cr-ME against these symptoms in this study, therefore, it is speculated that current effect of Cr-ME on LPS-induced lung injury model (Figure 5) and containing higher levels of numerous flavonoid compounds identified by LC-MS/MS spectrometric analysis (Figure 1i,j) seem to strongly contribute to its potential efficacy on various inflammatory symptoms and diseases in lung.

**Figure 6:**
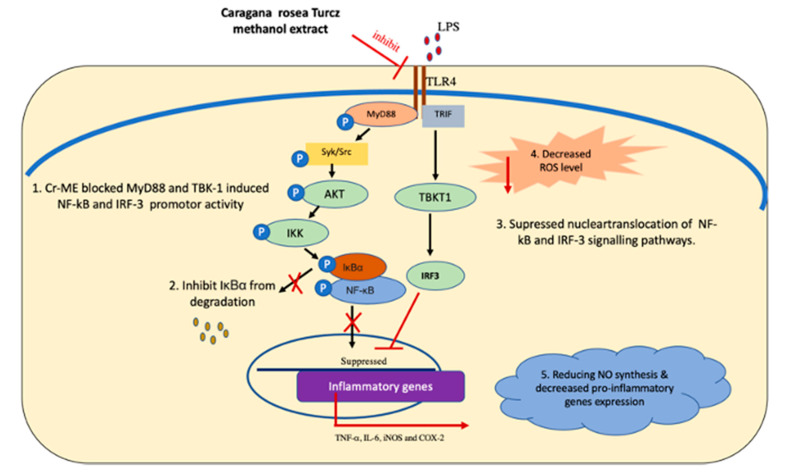
Anti-inflammatory mechanisms of Cr-ME targeting Src in the NF-κB and TBK1 in IRF3 signaling pathways.

In conclusion, we demonstrated that Cr-ME inhibits inflammatory processes, including NO production and mRNA expression of pro-inflammatory cytokines, both in vitro in LPS-induced RAW264.7 macrophages and in vivo in LPS-induced ALI in mice. The anti-inflammatory effects of Cr-ME could occur through a direct blockade of Src, which suppresses the NF-κB signaling cascades, as summarized in Figure 6. Therefore, Cr-ME is a potential herbal medicine that could be developed as a therapeutic anti-inflammatory remedy for prevention and treatment of inflammatory disease conditions.

## 4. Materials and Methods

### 4.1. Materials

Cr-ME was purchased from the Plant Diversity Research Center (DaeJeon, South Korea). Sodium dodecyl sulfate (SDS), 3-(4,5-dimethylthiazol,2-yl)-2,5-diphenyltetrazolium bromide (MTT), polyethylenimine (PEI), dimethyl sulfoxide (DMSO), polyinosinic: polycytidylic acid (Poly (I:C), and lipopolysaccharide (LPS, *Escherichia coli* O111:B4) were obtained from Sigma Chemicals Co. (St. Louis, MO, USA). Cell culture chemicals such as fetal bovine serum (FBS) was purchased from Biotechnics Research (Lake Forest, CA, USA), and Roswell Park Memorial Institute 1640 (RPMI 1640) and Dulbecco’s Modified Eagle Medium (DMEM) were obtained from HyClone (Grand Island, NY, USA). The RAW264.7 cell line (No. TIB-71) and human embryonic kidney cell line (HEK293T) (No. CRL-3216) were acquired from the American Type Culture Collection (ATCC, Rockville, MD, USA). Luciferase constructs with NF-κB and the PDR (III-I) luciferase promoter were used as previously reported [53]. Phospho- and total antibodies against p50, p65, Src, p-Src, Syk, p-Syk, IκBα, p-IκBα, IRF3, TBK1, and β-actin were purchased from Cell Signaling Technology (Beverly, MA, USA) and Santa Cruz Biotechnology (Santa Cruz, CA, USA).

### 4.2. Plant Collection, Specimen Information, and Cr-ME Preparation

Cr-ME (code number: FBM161-017) was acquired from International Biological Material Research Center (DaeJeon, Korea). The aerial part of the Cr-ME was collected and collection were done by the project team (Dr. Soo-Yong Kim). The aerial parts (leaves, shoot, and flowers) of *C. rosea* were collected, and dried in the shade and ground to a powder, which was used for the extraction process [54]. The extraction was performed using HPLC grade methanol on sonication (ultrasonic cleaner, BRANSON Ultrasonics Corporation, Brookfield, CT, USA), with 200 g of sample and 1.5 L of methanol at a temperature of 50 °C and sonication for 15 min, place for 2 h, followed by 3 days (10 times/day). Following extraction, the extract was filtered through a defatted cotton-plugged funnel to remove solid substances. The concentrate part of the filtered was completely under reduced pressure at 45 °C, followed by the residual amount weigh and the concentration of the solution was calculated. The extract solution dispensed by an equivalent of 20 mg per vial according to the concentration. The concentrated vial was completely under 50 °C and reduced pressure. After removal of the solvent under rescued pressure, the vial stored at −4 °C for long storage.

### 4.3. Cell Culture

RAW264.7 cells were maintained in RPMI 1640 supplemented with 10% heat-inactivated FBS and 1% antibiotics (penicillin-streptomycin). HEK293T cells were maintained in DMEM supplemented with 5% heat inactivated FBS. RAW264.7 and HEK293T cells were cultured at 37 °C and supplemented with 5% CO_2_ in a humidified incubator.

### 4.4. Drug Treatment

During the in vitro studies, the Cr-ME stock solution was made by dissolving the powdered, lyophilized Cr-ME stock with DMSO at a concentration of 100 mg/mL. When each experiment was performed, the stock solution was diluted to the desired final concentration of 50–200 μg/mL using the suitable culture medium. For the acute lung injury mouse model experiments, the stock of Cr-ME was prepared in 0.5% CMC (as a vehicle) and PBS, respectively, at doses of 100 mg/kg and 200 mg/kg. The doses and experimental scheme of Cr-ME in this study were determined, according to previously published papers showing similar in vitro and in vivo activities [55,56].

### 4.5. Determination of NO Production by Griess Assay

RAW264.7 cells were cultured overnight (1.2 × 10^6^ cells/mL), treated with Cr-ME (6.25–100 μg/mL) in a 96-well culture plate, then pre-treated with different concentrations of testing extract (Cr-ME) or compound (L-NAME) for 30 min. Inflammatory inducers [LPS (1 μg/mL), poly (I:C) (200 μg/mL), or pam3csk4 (10 μg/mL)] were then treated for additional 24 h [39]. The inhibitory effects of Cr-ME and L-NAME on NO production were detected using Griess reagents, as previously described [57]. Briefly, the suspension (100 μL) was mixed with 100 μL of Griess reagent, and the absorbance was analyzed at 540 nm. The NO concentration from culture supernatants was calculated by using the standard curve prepared with sodium nitrite (0 to 100 μM), as reported previously [58].

### 4.6. Cell Viability Assay (MTT)

The viability of RAW264.7 cells was determined using MTT assay [59]. After preincubation of the RAW264.7 cells (1.2 × 10^6^ cells/mL) for 18 h, the cells were treated with different concentrations of Cr-ME (50, 100, 150, and 200 μg/mL) and incubated for an additional 24 h. The cytotoxicity of the Cr-ME was evaluated using MTT assay as described previously [60]. Briefly, three hours prior to culture termination, 10 μL of MTT solution (10 mg/mL in PBS, pH 7.4) was added. The incubation was halted by adding 15% SDS to each well to solubilize the formazan. The absorbance at 570 nm (OD570-630) was measured using a Spectramax 250 microplate reader (BioTek, Bad Friedrichshall, Germany).

### 4.7. ROS Generation Assay

The 2′,7′-dihydrodichlorofluorescein diacetate (H2-DCFDA) assay was used to evaluate levels of ROS inside cells [61]. RAW264.7 cells were cultured at a density of 4 × 10^5^ cells/well in 12-well plates and irradiated with UV (30 mJ/cm^2^). Cells were incubated with Cs-ME (0, 50, and 100 μg/mL) or retinol (10 μg/mL) for 24 h. Cells were washed with cold PBS to slow metabolism and were stained with 50 μM H_2_-DCFDA stain for 30 min without exposure to light. Cells were fixed for 20 minutes and analyzed using an Eclipse Ti fluorescence microscope (Nikon, Tokyo, Japan). Mean fluorescence intensity (MFI) values were measured and the ROS generating cells were counted.

### 4.8. DPPH Assays

DPPH decolorimetric assays were performed to examine the scavenging of Cr-ME [62]. A mixture of Cr-ME (0–200 μM) and a mixture of 8-HD (0–50 μM) and 250 μM DPPH was incubated at 37 °C for 30 min. Ascorbic acid (500 μM) was used as a positive control. After incubation, the absorbance at 517 nm of each sample was measured by spectrophotometry. The DPPH scavenging effect was expressed as the percent inhibition:

DPPH scavenging effect (%) = [(A_0_ − A_1_)/A_0_] × 100

where A_0_ is the absorbance of DPPH, and A_1_ is the absorbance of the sample.

### 4.9. Liquid Chromatography Tandem Mass Spectrometry (LC-MS/MS)

To identify the phytochemical characteristics of Cr-ME, liquid chromatography tandem mass spectrometry (LC-MS/MS) was conducted as previously reported [63].

### 4.10. Gene Expression Determined by Semi-Quantitative RT-PCR and Quantitative Real-Time Polymerase Chain Reaction (qPCR) Analysis

Cytokine gene expression levels were determined in RAW264.7 cells. The cells were pretreated with Cr-ME (50–200 μg/mL) for 30 min and then stimulated with LPS (1 μg/mL) for 6 h. After 6h, the cells were harvested, and total RNA was isolated using TRIzol reagent (Gibco, Waltham, MA, USA) according to the manufacturer’s instructions. The isolated RNA was maintained at −70 °C until use. cDNA was synthesized using a cDNA synthesis kit (Thermo Fisher Scientific, Waltham, MA, USA) according to the manufacturer’s instructions. mRNA gene expression was determined using RT-PCR. The semiquantitative RT-PCR and qPCR were conducted as previously reported [64,65]. Primers were designed using online software and are listed in Table 2.

### 4.11. Luciferase Reporter Gene Assay

Plasmids (1 μg/mL) carrying the luciferase construct with gene promotors containing NF-κB and IRF3 binding sites were transfected along with the MyD88 and TBK1 adaptor genes into pre-plated HEK293T cells (1 × 10^6^ cells/mL). Transfection was performed using the PEI method overnight, and the transfected cells were treated with Cr-ME for 12 h. The luciferase activity was measured and calculated as described previously [66].

### 4.12. Protein Lysate Isolation, Nuclear Extracts and Western Blotting Analysis

RAW264.7 cells were seeded and incubated overnight, followed by pre-treatment with Cr-ME (100 μg/mL) for 30 min and treatment with LPS (1 μg/mL) for 5, 15, 30, and 60 min. The cells were harvested at those time points, and whole-cell protein lysates were prepared as previously described [22]. Nuclear lysates were prepared in a three-step procedure as reported previously [67]. The isolated protein samples were processed for Western blotting analysis. They were separated by SDS-polyacrylamide gel electrophoresis (PAGE) (Bio-Rad, Hercules, CA, USA) and then immunoblotted with specific total and phosphorylated antibodies as previously reported [40]. The bands were documented, and band intensity was measured using ImageJ software. For preparing lung lysate samples, the upper lobes of the right lungs of the mice used in the ALI experiment (see below) were collected, frozen in liquid nitrogen, and stored at −80 °C. The tissues were thawed and homogenized, and whole protein lysates were prepared as previously described [68].

### 4.13. Gene Overexpression

HEK293T cells were plated and incubated overnight before initiation of transfection. HA-SRC and FLAG-TBK1 genes were transfected using PEI for 24h to induce Src and TBK1 expression. The cells were treated with Cr-ME for 24 h. The protein samples were harvested, and transfection efficiency was determined using HA and FLAG antibodies. The total and phosphorylated Src, p50, p65, and TBK1 proteins were determined by Western blotting analysis.

### 4.14. Cellular Thermal Shift Assay (CETSA)

HEK293T cells were made to overexpress SRC using the PEI transfection method in a 6-well plate as described previously [56]. After 24 h, the transfected cells were treated with DMSO or Cr-ME (100 μg/mL) for an additional 24 h. Then, the cells were isolated and suspended in PBS. The cell suspension was divided into 10 equal volumes in PCR tubes (100 μL in each tube). The tubes were heated for 3 min in a temperature gradient from 35 °C to 71 °C and then cooled for 3 min at 25 °C. Subsequently, the cells were lysed using liquid nitrogen, followed by three repeated freeze–thaw cycles (thawing at 25 °C). The proteins were separated by centrifugation at 13,000 rpm for 30 min at 4 °C. The protein supernatant was analyzed using Western blotting analysis.

### 4.15. Lipopolysaccharide Induced Acute Lung Injury in Mice

C57BL/6 mice (age = 5-weeks, male, 17–20 g) were purchased from Orient-Bio (Sungnam, Korea). All animals were maintained in the animal holding facility of the institute under pathogen-free conditions. They were conditioned at 21–23 °C at a constant humidity of 40–60% and 12-h light–dark cycle and maintained on food and tritiated water *ad libitum*. The mice were divided into 5 study groups of 7 mice/group: control/vehicle (0.5% CMC) group, LPS (Sigma-Aldrich,) group, Cr-ME (50 mg/kg) + LPS group, Cr-ME (100 mg/kg) + LPS group, and DEXA (5 mg/kg) + LPS group. The mice in the control group were given only saline by oral administration using gavage needle. The mice in the Cr-ME + LPS groups were given the Cr-ME by oral gavage twice before 1 h of LPS treatment and once after LPS treatment. The mice in the DEXA + LPS group received DEXA by oral administration using gavage needle twice before 1 h of LPS treatment and once after LPS treatment. The animals in the LPS group were instilled intranasally with 5 mg/kg of LPS in saline twice, which is also how the other groups received their LPS treatments. After fasting for 24 h, the animals were sacrificed, and their lung tissues were collected aseptically.

### 4.16. Effects of Cr-ME on the Wet/Dry Lung Weight Ratio

The left lungs were excised, and then the lung tissues were washed thrice in normal saline, blotted dry, and weighed. After that, all the lung tissues were kept in an oven at 70 °C for 48 h, and then they were weighed again. The ratio of the wet-to-dry weight of each lung was calculated to measure pulmonary edema and to determine the protective effect of Cr-ME treatment.

### 4.17. Histological Analysis

The lower lobes of the right lungs of the mice used in the ALI experiment were excised, placed in 4% paraformaldehyde, and kept overnight for 24 h fixation. Then the samples were embedded in paraffin, sectioned at 5 µm, and stained with hematoxylin and eosin. The lung tissue was examined histopathologically under a brightfield microscope (Optinity microscope, #KI-3000F, company MDM instruments, Suwon, Korea) and analyzed using the Optiview software. The lung injury score was performed based on the American Thoracic Society’s ALI scoring system [69].

### 4.18. Statistical Analysis

All data are presented as mean ± standard deviation (SD). All statistical analyses were performed using Sigma plot (Chicago, IL, USA) and Graph Pad Prism version 6.01 software (San Diego, CA, USA). Statistical comparisons were performed using one-way ANOVA (Dunnett’s *t*-test). *p* values < 0.05 were considered statistically significant.

## 5. Conclusions

We have demonstrated that Cr-ME inhibits inflammatory processes, including NO production and mRNA expression of pro-inflammatory cytokines, both in vitro in LPS-induced RAW264.7 macrophages and in vivo in LPS-induced ALI in mice. The anti-inflammatory effects of Cr-ME could occur through a direct blockade of Src, which suppresses the NF-κB signaling cascades, as summarized in Figure 6. Therefore, Cr-ME is a potential herbal medicine that could be developed as a therapeutic anti-inflammatory remedy for prevention and treatment of inflammatory disease conditions.

## Figures and Tables

**Table 1:** Lung injury scoring system.

Parameters	Scoring per Field		
	0	1	2
**Neutrophils in the alveolar space**	none	1 to 5	>5
**Neutrophils in the interstitial space**	none	1 to 5	>5
**Number of hyalin membrane**	none	1	>1
**Amount of proteinaceous debris filling in the airspace**	none	1	>1
**Alveolar septal thickening**	<2×	2×–4×	>4×

Score = [(20 × A) + (14 × B) + (7 × C) + (7 × D) + (2 × E)]/(number of fields × 100).

**Table 2:** Primers used in this study (mouse species).

Targets	Direction	Sequences (5′ to 3′)
iNOS	Forward Reverse	GTGAAGAAAACCCCTGTGCTG AGTTCCGAGCGTCAAAGACC
TNF-α	Forward Reverse	TGCCTATGTCTCAGCCTCTT GAGGCCATTTGGGAACTTCT
IL-6	Forward Reverse	GCCTTCTTGGGACTGATGCT TGGAAATTGGGGTAGGAAGGAC
COX-2	Forward Reverse	CATCCCCTTCCTGCGAAGTT CATGGGAGTTGGGCAGTCAT
MMP-2	Forward Reverse	TCCCTGGAGACCTGAGAACC GGCAAGTCTTCCGAGTAGTTT
MMP-9	Forward Reverse	GCCACTTGTCGGCGATAAGG CACTGTCCACCCCTCAGAGC
IFN-β	Forward Reverse	GGCCTTGGGCCTCAAAGGAA GCTTGGGATCCACACTCTCCA
GAPDH	Forward Reverse	ACCACAGTCCATGCCATCAC CCACCACCCTGTTGCTGTAG

## Data Availability

The data used to support the findings of this study are available from the corresponding author upon request.

## Abbreviations

TAK1	Transforming growth factor-β-activated kinase 1
NF-κB	Nuclear factor-κB
AP-1	Activator protein-1
MAPKs	Mitogen-activated protein kinases
JNK	c-Jun N-terminal kinase
IRF3	Interferon regulatory factor 3
PI3K	Phosphoinositide 3-kinase
PGE_2_	Prostaglandin E_2_
COX-2	Cyclooxygenase-2
IL-1β	Interleukin-1β
IL-6	Interleukin 6
TNF-α	Tumor necrosis factor alpha
MKK	Mitogen-activated protein kinase
iNOS	Inducible nitric oxide synthase
